# A complicated infection by cutaneous *Nocardia wallacei* and pulmonary *Mycobacterium abscessus* in a Chinese immunocompetent patient: a case report

**DOI:** 10.3389/fcimb.2023.1229298

**Published:** 2023-08-16

**Authors:** Ling Qin, Sidan Wang, Zhifen Zheng, Wenqian Zhang, Qiang Qu, Jun Li, Yurong Tan, Liming Cao

**Affiliations:** ^1^ Department of Respiratory Medicine, National Key Clinical Specialty, Branch of National Clinical Research Center for Respiratory Disease, Xiangya Hospital, Central South University, Changsha, Hunan, China; ^2^ Clinical Research Center for Respiratory Diseases, Xiangya Hospital, Changsha, Hunan, China; ^3^ National Clinical Research Center for Geriatric Disorders, Xiangya Hospital, Changsha, Hunan, China; ^4^ BGI Genomics, Shenzhen, China; ^5^ Clinical Laboratories, BGI Genomics, Wuhan, China; ^6^ Department of Laboratory Medicine, Xiangya Hospital, Central South University, Changsha, Hunan, China; ^7^ Department of Medical Microbiology, School of Basic Medical Sciences, Central South University, Changsha, Hunan, China

**Keywords:** *Nocardia wallacei*, cutaneous Nocardiosis, *Mycobacterium abscessus*, mNGS, mixed infection

## Abstract

Nocardiosis is an infectious disease caused by *Nocardia* that primarily affects immunocompromised hosts. *Mycobacterium abscessus* is a common opportunistic pathogen that causes disease in humans, including pulmonary and extrapulmonary infection. *Nocardia* spp. infection is uncommon, and infection with *Nocardia wallacei* and *Mycobacterium abscessus* is even rarer. A 59-year-old immunocompetent woman with risk factors for environmental exposure developed nocardiosis and presented to the hospital with a cough, shortness of breath, hemoptysis, and a back abscess. An enhanced computed tomography (CT) of the chest revealed partial destruction of the right lung, as well as consolidation of the right upper lobe. Rare pathogens *N. wallacei* and *Mycobacterium abscessus* were detected by metagenomic next-generation sequencing (mNGS) from abscess on the back and lung puncture tissue, respectively. She was treated with a combination of antibiotics and was finally discharged with a good prognosis. In this case, we present a patient who was successfully diagnosed with *N. wallacei* and *Mycobacterium abscessus* infection using mNGS. This importance of using mNGS in pathogen detection and the effective use of antibiotics in treating patients with long-term rare infections is highlighted in this report.

## 1 Introduction

*Nocardia* spp. are aerobic, Gram-positive filamentous branching bacteria with a slow growth rate and a partial acid tolerance. *Nocardia* spp. are uncommon in dust, decaying vegetation, soil, and aquatic environments(Chen et al., 2014; Wang et al., 2015). Over 80 species of *Nocardia* have been described, with approximately 30 of them known to cause human disease(King et al., 2009). Nocardiosis primarily affects immunocompromised patients, though immunocompetent individuals may be affected in rare cases. HIV infection, inflammatory bowel disease, chronic lung disease, solid-organ transplantation, autoimmune diseases, corticosteroids use and hematological malignancy are major risk factors for nocardia infection.

Because *Nocardia* spp. is most commonly transmitted through inhalation, the lung is the most common site of infection (62-86%)(Minero et al., 2009; Haussaire et al., 2017). In approximately 8-31% of patients with invasive nocardiosis, skin involvement can take the form of a single or cluster of pustules, nodules, or deep-seated abscesses, which may also involve the muscles(Coussement et al., 2016; Haussaire et al., 2017). Primary cutaneous nocardiosis can develop in immunocompetent patients as a result of direct microorganism inoculation into the skin as a result of trauma, which is most common in rural agricultural workers(Kanne et al., 2011; Lafont et al., 2020).

*Mycobacterium abscessus* is an important nontuberculous mycobacterium (NTM) that causes disease in humans, which is a common opportunistic pathogen(Boudehen and Kremer, 2021). *Mycobacterium abscessus* infections typically affect the skin, soft tissues, and lungs, although they can also occur in other parts of the body. In lung infections, symptoms can include persistent cough, shortness of breath, chest pain, and coughing up blood. The prevalence of *M. abscessus* infections vary geographically(Hsu et al., 2022). It has been reported to be more common in certain regions, such as the southeastern United States, where hot and humid climates provide favorable conditions for the growth of these bacteria(Dahl et al., 2022).

Direct examination is used to diagnose nocardiosis, which is primarily based on pathogen culture and Gram staining. In contrast, *Nocardia* requires at least 2-7 days of culture to slow growth and up to 4-6 weeks to develop into visible colonies(Williams et al., 2020). Metagenomic next-generation sequencing (mNGS) is a culture-independent method for detecting infectious pathogens, particularly rare or novel pathogens, and it outperforms traditional diagnostic methods, indicating its potential for use in early diagnosis(Li et al., 2021). Furthermore, mNGS takes only about 24 hours, allowing for rapid pathogen identification in the case of complex infections.

In this case, we present a patient who was successfully diagnosed with *N. wallacei* and *Mycobacterium abscessus* infection using mNGS. In the current literature, there are no reported cases of host infection with both bacteria simultaneously. This is the first case of immunocompetent patient with both *Nocardia wallacei* and *Mycobacterium abscessus* infection in China, and its clinical features and treatment experience have important value for the use of antibiotics and the treatment of pulmonary infection.

## 2 Case representation

A 59-year-old woman was presented to our hospital in April 2022 with a 6-year history of coughing, shortness of breath, and a back lump, as well as a 5-year history of hemoptysis that had worsened for 3 months. The cough was paroxysmal, with mucus sputum and pulling pain in the chest and back. Over the preceding 6 years, she had noticed a lump on her right back with mild pain and a clear border. Previously She had previously been admitted to the local hospital and had received anti-infection treatment. With irregular treatment in 6 years, the size of the lump was not fixed. There was no drainage from the abscess, and she had no history of similar skin lesions. In addition to bronchiectasis, respiratory failure, pulmonary heart disease, and pulmonary hypertension, she has chronic obstructive pulmonary disease (COPD). Prior to this presentation, she was taking sulbactam, cefopcrazone, and meropenem in combination with Moxifloxacin for infection control. Over the last three months, the pain in her right chest and back has worsened, and the lump on her back has grown larger. She had been on glucocorticoids for pain relief for the previous month. The patient has no family history of pulmonary and cutaneous infections. In the history of trauma, the patient had a fracture in the distal right radius in 2016, but without particular discomfort after recovery.

**Figure 1:**
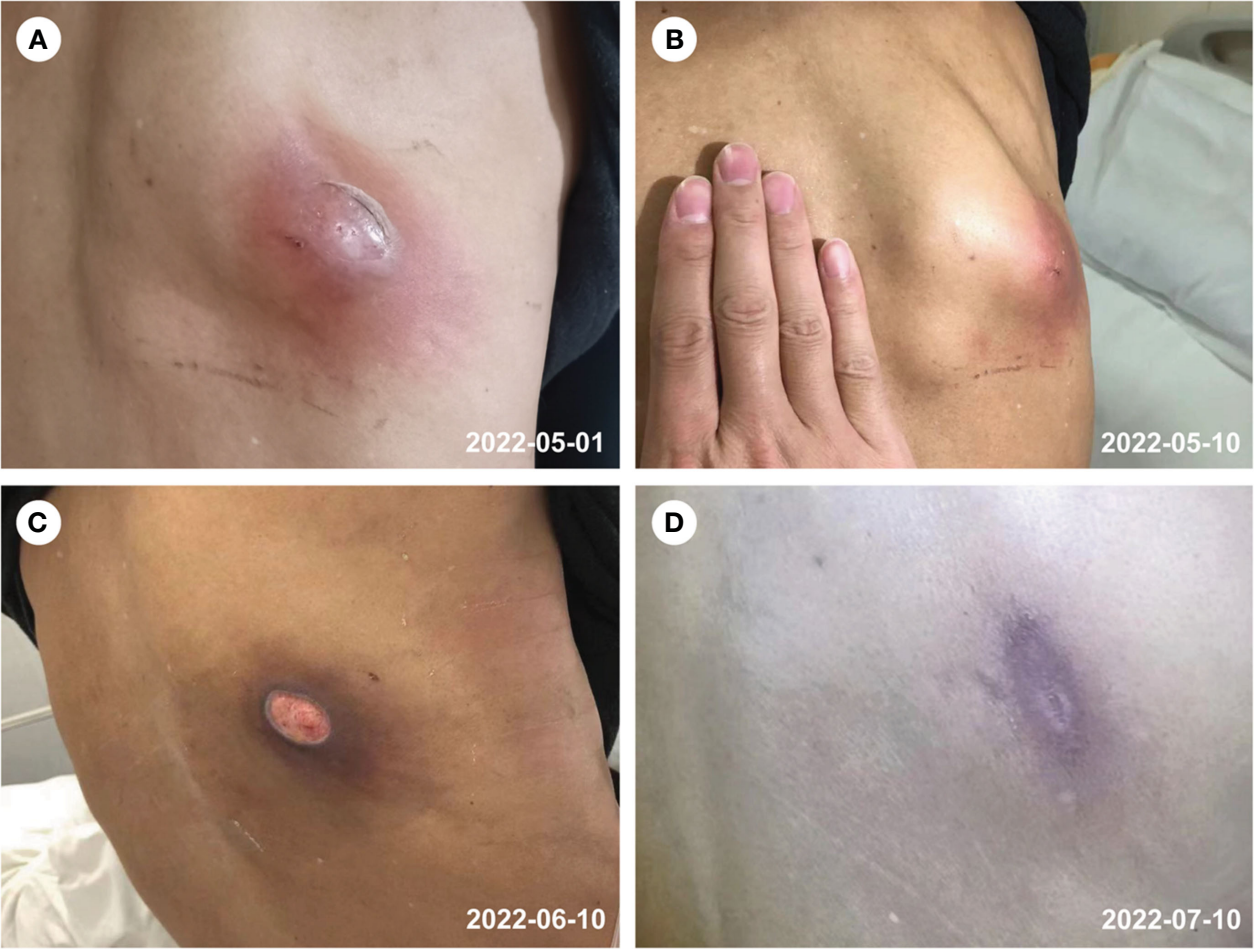
Abscess of the right back **(A–D)**. **(A)** prior to the treatment, **(B)** after 2 weeks of treatment, **(C)** after 6 weeks of treatment, **(D)** follow-up on 10th July 2022.

When the patient arrived, her vital signs were normal and she was conscious. There was no thoracic deformity. A 7*4 cm lump can be seen on the right back, which is soft and fluctuating, with clear border and pressure pain (**Figure 1**). The patient’s breath sounds were weakened on the right chest, while vocal conduction and moist rales were emphasized. The cardiovascular and nervous systems performed admirably.

**Figure 2:**
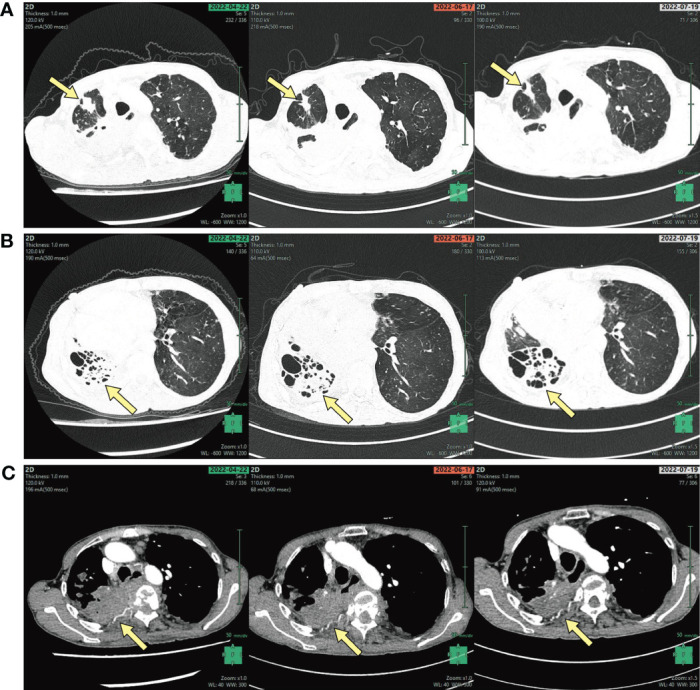
**(A)** CT of the chest shows a right upper lung nodule, **(B)** destruction of the right lung, **(C)** the bone destruction of the right ribs (arrows, **A–C**).

The hemoglobin concentration was 83 g/L, the HCT was 27.1%, and the erythrocyte sedimentation rate (ESR) was 120mm/h, according blood tests. The tumor markers were all negative. An enhanced computed tomography (CT) of the chest revealed a right shift of the mediastinum and partial destruction of the right lung, as well as consolidation of the right upper lobe (**Figure 2**). An ultrasound of the heart revealed an enlarged right ventricle and pulmonary hypertension. The SPAP was calculated to be 69 mmHg. Magnetic resonance imaging (MRI) of the brain showed high signals in the paraventricular white matter, which could be vasogenic.

Tissue samples from the back abscess and lung samples were sent for pathological examination by metagenomic next-generation sequencing (mNGS) (Beijing Genomics Institute (BGI)-Wuhan, China). The mNGS was performed in BGI-Wuhan exactly following the previous protocol, including DNA extraction, libraries construction and sequencing. High-quality sequencing data were generated by removing low-quality reads, followed by computational subtraction of human host sequences mapped to the human reference genome (hg19) using Burrows-Wheeler Alignment. The remaining data were classified by simultaneously aligning to Pathogens metagenomics Database (PMDB), consisting of bacteria, fungi, viruses and parasites.

Samples from the back abscess and the lung tissue identified the pathogenic pathogen infection. In the back abscess, acid-fast staining, X-pert, and PPD (tuberculin pure protein derivative) test were all negative, while mNGS pathological examination showed *N. wallacei* from the tissue samples, and mNGS of the blood sample also identified the same result Regarding the pulmonary infection, sputum, lung tissue, and blood sample were used to identify *M. abscessus* infection. The aerobic culture and swab of sputum revealed moderate G+ coccus and mild G+ bacillus. The mNGS of lung samples showed *M. abscessus* infection. The lungs’ pathology revealed fibrous tissue proliferation but no evidence of malignancy. In this case, blood and abscess samples were collected from patients on April 29, 2022 and May 11, 2022, respectively, for mNGS detection (**Table 1**). After treatment, the RPM (reads per million sequences) of *N. wallacei* in abscess decreased from 795.12 to 239.10 by NGS.

**Table 1:** Next-generation sequencing of abscess and blood samples for the patient.

Sample typle	Date of sample collection	Total Reads	Mapped to bacteria reads	Latin name of strain	standardized unique reads	RPM	Coverage Rate(%)
Abscess	2022/4/29	42,140,853	55,007	Nocardia wallacei	33,507	795.12	19.62
Lung tissue	2022/4/29	97,536,910	21,085	Mycobacteroides abscessus	13	0.13	0.01
Abscess	2022/5/11	92,657,184	52,793	Nocardia wallacei	22,154	239.10	13.29
Blood	2022/5/11	53,108,020	58,751	Nocardia wallacei	131	2.47	0.09

Based on the pathogen detection and clinical symptoms, she was diagnosed with pulmonary infection caused by *Mycobacterium abscessus* and cutaneous nocardiosis caused by *N. wallacei*. Before the pathogenic pathogen was confirmed, she was started on Piperacillin and Tazobactam 4.5g every 8 hours for 9 days for empiric anti-infection treatment. After the diagnosis, her treatment was changed to Imipenem 1.0g ivgtt, Clarithromycin 0.5g po, Doxycycline 0.1g po every 12 hours to suppress *Mycobacterium abscessus*, and TMP-SMX 0.96g po every 6 hours to suppress *N. wallacei*. Twelve days after treatment, her anti-infection therapy was changed to a combination of SMX, Doxycycline, Clarithromycin, and Cefoxitin. which is recommended as a long-term treatment. The patient’s condition has improved with her back abscess significantly become smaller as a result of targeted medications, and she has returned to the local hospital with the treatment plan for further observation. A follow-up CT of the chest two months later revealed that most of the right lung lesion had been absorbed miraculously, and her clinical symptoms had improved with a favorable prognosis.

## 3 Dissussion

We present a case of a patient who was successfully diagnosed with *N. wallacei* and *Mycobacterium abscessus* infection using mNGS. This is the first case of infection with both *N. wallacei* and *Mycobacterium abscessus* in an immunocompetent patient in China. Although inhalation is the most common clinical manifestation of nocardiosis, skin contact with the bacteria can result in extrapulmonary dissemination. In this case, the patient has a cutaneous *N. wallacei* infection as well as a pulmonary *Mycobacterium abscessus* infection.

Infections caused by *Nocardia* spp. are becoming more common, but the infection caused by *N. wallacei* was limited. The first human infection of *N. wallacei* was reported in the United States in 1979. At that time, it was identified as the drug pattern IV of *N. asteroides*(Conville et al., 2008). In 2008, Conville et al. proposed *N. wallacei* as a new species designation to the drug pattern IV, which was recognized with the name *N. wallacei*(Conville et al., 2008). In China, the first *N. wallacei* infection reported in an immunocompetent patient with pulmonary nocardiosis and without disseminated infection. There have been additional reports of disseminated infection in the United States, France, and Mexico(Cassir et al., 2013; Cooper et al., 2014; Welsh et al., 2018).

**Figure 3:**
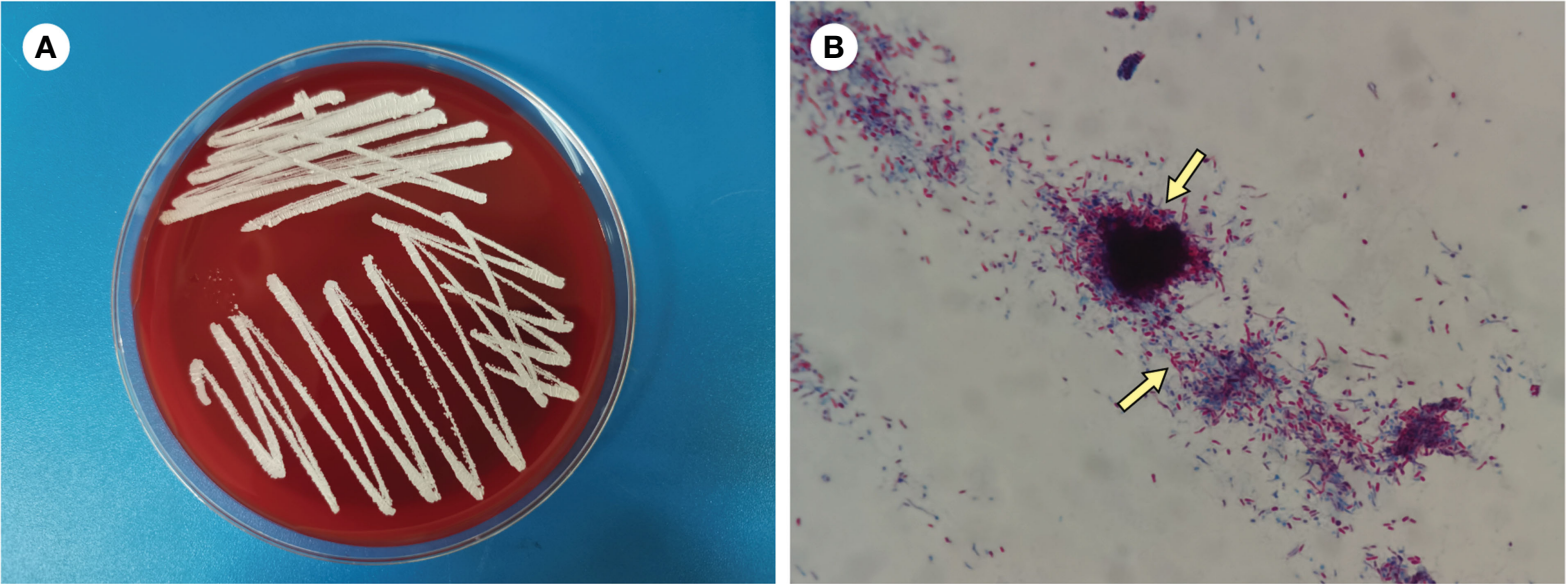
**(A)** Culture on blood agar plate for 3 days **(B)** Modified acid-fast staining of BALF (×1000).

*Nocardia wallacei* and *Mycobacterium abscessus* are both opportunistic pathogenic bacteria that can cause infections, particularly in immunocompromised individuals(Boudehen and Kremer, 2021). Patients with weakened immune system, such as those with diabetes, ulcerative colitis, cirrhosis, HIV infection, and stem cell or solid organ transplant recipients, are more likely to contract *N. wallacei* infections (Puing et al., 2021). The effects of these infections in immunocompromised patients can be significant and potentially severe. In both *Nocardia wallacei* and *Mycobacterium abscessus* infections, the immunocompromised state of the patient can contribute to increased susceptibility, more severe infections, and a higher risk of complications. However, our case shows infection of *N. wallecei* and *M. abscessus* in an immunocompetent patient, making this the first instance of these two pathogens co-infecting. The three-day cultivation results confirmed the mNGS results (**Figure 3**).

It may be difficult to diagnose such patients with pulmonary and extrapulmonary infection. Before the pathogens were identified, she was given anti-infection treatments several times, but she did not respond well. Conventional specimen evaluation and primary diagnostic methods, such as bacterial culture of abscess punctual fluid, sputum culture and Acid-Fast Bacteria (AFB) Staining, were all negative. Pathological examination of lung tissue samples revealed no evidence of malignancy. Although molecular biology with amplification and sequencing of one or two genes among rrs, hsp65, secA1 and soda (Margalit et al., 2021)is the gold standard for Nocardia species identification, it is limited by detection time and equipment availability. The mNGS, an emerging diagnostic method, has excelled in the rapid clinical diagnosis of a variety of diseases. When compared to traditional serology and culture methods, it has a shorter turnaround time, higher accuracy, and better diagnostic performance(Miao et al., 2018). Our findings suggested that mNGS could provide a method of monitoring disease progression and therapeutic efficacy. The successful diagnosis of rare pathogen infection using mNGS in this case suggests a more effective and straightforward way to identify rare pathogen infection, which contributes to the successful anti-infection treatment.

*N. wallacei* is expected to be toxic to sulfonamide antibiotics, particularly trimethoprim-sulfamethoxazole (TMP-SMX). TMP-SMX remains the gold standard, with active ingredients include imipenem, linezolid, ceftriaxone, and fluoroquinolones(Derungs et al., 2021). Amikacin, imipenem, or meropenem should be given in addition to TMP-SMX in immunocompromised and disseminated infections caused by *N. wallacei*(Bryant et al., 2021). Local abscesses, on the other hand, are typically treated with incision, drainage, and surgery to remove necrotic tissue(Weng et al., 2020). For *Mycobacterium abscessus* infection, susceptibility-based treatment for macrolides and amikacin is preferred over empiric therapy for *Mycobacterium abscessus* infection(Nick et al., 2022), and its active drugs include clarithromycin, azithromycin, imipenem, cefoxitin, and tigecycline based on *in vitro* susceptibility test results. The 2020 NTM Guideline recommended a multidrug treatment regimen containing at least 3 active antibiotics to treat its pulmonary infection(Daley et al., 2020). Antibiotic susceptibility should be tested prior to treatment and antibiotic combination therapy should be chosen based on the susceptibility test results(Griffith and Daley, 2022).

To treat the *Norcadosis* and *Mycobacterium* infection, a combination of TMP-SMX and imipenem, clarithromycin, and doxycycline was used for a total of 12 days. Her clinical improvement was linked to the phage treatment. After the initial period of infection treatment, we switched to a combination of TMP-SMX, Doxycycline, Clarithromycin, and Cefoxitin, which is recommended as a long-term treatment. Her follow-up exams revealed that the abscess on her back had been absorbed, and her pulmonary lesions had significantly improved.

Because Nocardiosis has a high mortality rate and a high rate of misdiagnosis(Soueges et al., 2022), early diagnosis and treatment are critical, especially infection with other pathogens. In our case, the patient began empiric antibiotic treatment a year before the pathogen was identified, but it had little effect. Her pre-existing medical conditions may worsen as a result of the delay in administering proper antimicrobials. The application of mNGS in pathogen detection contributes significantly to the case’s successful diagnosis and treatment. The treatment of *N. wallacei* infection requires early and accurate pathogen diagnosis of Nocardiosis.

*Nocardia wallacei* and *Mycobacterium abscessus* are both opportunistic pathogens. Only one third of infections caused by *Nocardia wallacei* happen in immunocompetent patients, while hosts infected by *Mycobacterium abscessus* are mostly immunocompromised(Johansen et al., 2020; Sur et al., 2022). Infections caused by these two bacteria alone are rare in immunocompetent patients, and the simultaneous infection is even rarer(Wei et al., 2021). This case is the first successfully treated infection with *N. wallacei* and *M. abscessus* in China. The treatment process and medication regimen have important value for the treatment of rare pathogen infection, especially the treatment of these infection in immunocompetent patients.

## 4 Conclusion

This is the first case of *N. wallacei* and *M. abscessus* simultaneous infection in an immunocompetent patient in China. mNGS technology was used to confirm the pathogens, and the patient was discharged with her condition improved after timely and proper anti-infection treatment. This case shows that mNGS has a significant advantage in the detection of rare and mixed infections. In this case, treating a rare disseminated infection successfully can help clinicians deal with rare infections by preventing underdiagnosis and misdiagnosis.

## Data availability statement

The datasets presented in this study are deposited in online repositories. The names of the repository/repositories and accession number(s) can be found below: https://www.ncbi.nlm.nih.gov/sra/PRJNA982594.

## Ethics statement

Written informed consent was obtained from the individual(s) for the publication of any potentially identifiable images or data included in this article. Written informed consent was obtained from the participant/patient(s) for the publication of this case report.

## Author contributions

LQ and LC conceived the study. LQ drafted the first manuscript. SW, QQ, JL and YT followed the patients during the diagnostic and therapeutic path. ZZ and WZ provided data analysis and interpretation for the mNGS detection. All authors contributed to the article and approved the submitted version.

## References

[B1] Boudehen Y.-M. Kremer L. (2021). Mycobacterium abscessus. Trends Microbiol. 29 (10), 951–952. doi: 10.1016/j.tim.2021.06.006 34312062

[B2] Bryant J. M. Brown K. P. Burbaud S. Everall I. Belardinelli J. M. Rodriguez-Rincon D. . (2021). Stepwise pathogenic evolution of Mycobacterium abscessus. Science 372 (6541), eabb8699. doi: 10.1126/science.abb8699 33926925 PMC7611193

[B3] Cassir N. Million M. Noudel R. Drancourt M. Brouqui P. (2013). Sulfonamide resistance in a disseminated infection caused by Nocardia wallacei: a case report. J. Med. Case Rep. 7 (1), 1–4. doi: 10.1186/1752-1947-7-103 23577983 PMC3633055

[B4] Chen J. Zhou H. Xu P. Zhang P. Ma S. Zhou J. (2014). Clinical and radiographic characteristics of pulmonary nocardiosis: clues to earlier diagnosis. PloS One 9 (3), e90724. doi: 10.1371/journal.pone.0090724 24594890 PMC3940923

[B5] Conville P. S. Brown J. M. Steigerwalt A. G. Brown-Elliott B. A. Witebsky F. G. (2008). Nocardia wallacei sp. nov. and Nocardia blacklockiae sp. nov., human pathogens and members of the “Nocardia transvalensis Complex”. J. Clin. Microbiol. 46 (4), 1178–1184. doi: 10.1128/JCM.00994-11 18256227 PMC2292930

[B6] Cooper C. J. Said S. Popp M. Alkhateeb H. Rodriguez C. Aguilar M. P. . (2014). A complicated case of an immunocompetent patient with disseminated nocardiosis. Infect. Dis. Rep. 6 (1), 5327. doi: 10.4081/idr.2014.5327 24757510 PMC3987247

[B7] Coussement J. Lebeaux D. van Delden C. Guillot H Freund R Marbus S . (2016). Nocardia infection in solid organ transplant recipients: a multicenter European case-control study. Rev. Infect. Dis. 63 (3), 338–345. doi: 10.1093/cid/ciw241 27090987

[B8] Dahl V. N. Mølhave M. Fløe A. van Ingen J. Schön T. Lillebaek T. . (2022). Global trends of pulmonary infections with nontuberculous mycobacteria: a systematic review. Int. J. Infect. Dis. 125, 120–131. doi: 10.1093/cid/ciaa241 36244600

[B9] Daley C. L. Iaccarino J. M. Lange C. Cambau E. Wallace R. J. Jr. Andrejak C. . (2020). Treatment of nontuberculous mycobacterial pulmonary disease: an official ATS/ERS/ESCMID/IDSA clinical practice guideline. Clin. Infect. Dis. 71 (4), e1–e36. doi: 10.1093/cid/ciaa241 32628747 PMC7768748

[B10] Derungs T. Leo F. Loddenkemper C. Schneider T. (2021). Treatment of disseminated nocardiosis: A host–pathogen approach with adjuvant interferon gamma. Lancet Infect. Dis. 21 (10), e334–e340. doi: 10.1016/S1473-3099(20)30920-8 34425068

[B11] Griffith D. E. Daley C. L. (2022). Treatment of Mycobacterium abscessus pulmonary disease. Chest 161 (1), 64–75. doi: 10.1164/rccm.200604-571ST 34314673

[B12] Haussaire D. Fournier P.-E. Djiguiba K. Moal V. Legris T. Purgus R. . (2017). Nocardiosis in the south of France over a 10-years period 2004–2014. Int. J. Infect. Dis. 57, 13–20. doi: 10.1016/j.ijid.2017.01.005 28088585

[B13] Hsu J.-Y. Cheng A. Ku C.-C. Chen Y.-C. Wang J.-T. Hsieh T.-W. . (2022). Mycobacterium abscessus and Mycobacterium massiliense exhibit distinct host and organ specificity: a cross-sectional study. Int. J. Infect. Dis. 116, 21–26. doi: 10.1016/j.ijid.2021.12.348 34954310

[B14] Johansen M. D. Herrmann J.-L. Kremer L. (2020). Non-tuberculous mycobacteria and the rise of Mycobacterium abscessus. Nat. Rev. Microbiol. 18 (7), 392–407. doi: 10.1038/s41579-020-0331-1 32086501

[B15] Kanne J. P. Yandow D. R. Mohammed T.-L. H. Meyer C. A. (2011). CT findings of pulmonary nocardiosis. Am. J. Roentgenology 197 (2), W266–W272. doi: 10.2214/AJR.10.6208 21785052

[B16] King A. S. Castro J. G. Dow G. C. (2009). Nocardia farcinica lung abscess presenting in the context of advanced HIV infection: Spontaneous resolution in response to highly active antiretroviral therapy alone. Can. J. Infect. Dis. Med. Microbiol. 20 (3), e103–e106. doi: 10.1155/2009/181750 20808449 PMC2770310

[B17] Lafont E. Conan P.-L. Rodriguez-Nava V. Lebeaux D. (2020). Invasive nocardiosis: disease presentation, diagnosis and treatment–old questions, new answers? Infection Drug Res. 13, 4601–4613. doi: 10.2147/IDR.S249761 PMC7764858 33376366

[B18] Li N. Cai Q. Miao Q. Song Z. Fang Y. Hu B. (2021). High-throughput metagenomics for identification of pathogens in the clinical settings. Small Methods 5 (1), 2000792. doi: 10.1002/smtd.202000792 33614906 PMC7883231

[B19] Margalit I. Lebeaux D. Tishler O. Goldberg E. Bishara J. Yahav D. . (2021). How do I manage nocardiosis? Clin. Microbiol. Infection 27 (4), 550–558. doi: 10.1016/j.cmi.2020.12.019 33418019

[B20] Miao Q. Ma Y. Wang Q. Pan J. Zhang Y. Jin W. . (2018). Microbiological diagnostic performance of metagenomic next-generation sequencing when applied to clinical practice. Clin. Infect. Dis. 67 (suppl_2), S231–S240. doi: 10.1093/cid/ciy693 30423048

[B21] Minero M. V. Marín M. Cercenado E. Rabadán P. M. Bouza E. Muñoz P. (2009). Nocardiosis at the turn of the century. Medicine 88 (4), 250–261. doi: 10.1097/MD.0b013e3181afa1c8 19593231

[B22] Nick J. A. Dedrick R. M. Gray A. L. Vladar E. K. Smith B. E. Freeman K. G. . (2022). Host and pathogen response to bacteriophage engineered against Mycobacterium abscessus lung infection. Cell 185 (11), 1860–1874, e1812. doi: 10.1016/j.cell.2022.04.024 35568033 PMC9840467

[B23] Puing A. G. Epstein D. J. Banaei N. Subramanian A. K. Liu A. Y. (2021). Nocardiosis in immunocompromised patients on alternative pneumocystis prophylaxis. Emerging Infect. Dis. 27 (10), 2734. doi: 10.3201/eid2710.210620 PMC8462344 34545802

[B24] Soueges S. Bouiller K. Botelho-Nevers E. Gagneux-Brunon A. Chirouze C. Rodriguez-Nava V. . (2022). Prognosis and factors associated with disseminated nocardiosis: a ten-year multicenter study. J. Infection 85 (2), 130–136. doi: 10.3201/eid2710.210620 35654278

[B25] Sur S. Patra T. Karmakar M. Banerjee A. (2022). Mycobacterium abscessus: insights from a bioinformatic perspective. Crit. Rev. Microbiol. 49 (4), 499–514. doi: 10.1080/1040841X.2022.2082268 35696783

[B26] Wang H.-K. Sheng W.-H. Hung C.-C. Chen Y.-C. Lee M.-H. Lin W. S. . (2015). Clinical characteristics, microbiology, and outcomes for patients with lung and disseminated nocardiosis in a tertiary hospital. J. Formosan Med. Assoc. 114 (8), 742–749. doi: 10.1016/j.jfma.2013.07.017 24008153

[B27] Wei M. Xu X. Yang J. Wang P. Liu Y. Wang S. . (2021). MLSA phylogeny and antimicrobial susceptibility of clinical Nocardia isolates: a multicenter retrospective study in China. BMC Microbiol. 21 (1), 1–11. doi: 10.1186/s12866-021-02412-x 34903163 PMC8667443

[B28] Welsh O. Salinas-Carmona M. C. Brown-Elliott B. A. Smith T. Cardenas-De La Garza J. A. Wallace J. R. J. (2018). Disseminated actinomycetoma due to Nocardia wallacei. Int. J. Dermatol. 57 (5), 580–582. doi: 10.1111/ijd.13909 29399787

[B29] Weng Y.-W. Huang C.-K. Sy C.-L. Wu K.-S. Tsai H.-C. Lee S. S.-J. (2020). Treatment for Mycobacterium abscessus complex–lung disease. J. Formosan Med. Assoc. 119, S58–S66. doi: 10.1016/j.jfma.2020.05.028 32527504

[B30] Williams E. Jenney A. W. Spelman D. W. (2020). Nocardia bacteremia: a single-center retrospective review and a systematic review of the literature. Int. J. Infect. Dis. 92, 197–207. doi: 10.1016/j.ijid.2020.01.011 31978577

